# Not all that looks fractured is broken—multipartite humeral epicondyles in children

**DOI:** 10.1007/s00330-022-08670-1

**Published:** 2022-03-17

**Authors:** Andrea Schmid, Anna-Maria Lois, Corona Metz, Jan-Peter Grunz, Simon Veldhoen

**Affiliations:** 1 Department of Diagnostic and Interventional Radiology, University Hospital Würzburg, Oberdürrbacher Straße 6, 97080 Würzburg, Germany; 2 Department of Radiology - Pediatric Radiology, Charité – Universitätsmedizin Berlin, corporate member of Freie Universität Berlin and Humboldt-Universität zu Berlin, Augustenburger Platz 1, 13353 Berlin, Germany

**Keywords:** Elbow joint, Epicondyles, Bone fractures, Pediatrics, Radiography

## Abstract

**Objective:**

Multipartite epicondyles may mimic fractures in the setting of pediatric elbow trauma. This study examines the prevalence of multipartite epicondyles during skeletal development and their association with pediatric elbow fractures.

**Materials and methods:**

In this retrospective analysis, 4282 elbow radiographs of 1265 elbows of 1210 patients aged 0–17 years were reviewed. The radiographs were analyzed by two radiologists in consensus reading, and the number of visible portions of the medial and lateral epicondyles was noted. For elbows in which epicondylar ossification was not yet visible, the epicondyles were already fused with the humerus or could not be sufficiently evaluated due to projection issues or because osteosynthesis material was excluded. In total, 187 elbows were included for the lateral and 715 for the medial epicondyle analyses.

**Results:**

No multipartite medial epicondyles were found in patients without history of elbow fracture, whereas 9% of these patients had multipartite lateral epicondyles (*p* < 0.01). Current or previous elbow fractures increased the prevalence of multipartite epicondyles, with significant lateral predominance (medial epicondyle + 9% vs. lateral + 24%, *p* < 0.0001). Including all patients regardless of a history of elbow fracture, multipartite medial epicondyles were observed in 3% and multipartite lateral epicondyles in 18% (*p* < 0.0001). There was no gender difference in the prevalence of multipartition of either epicondyle, regardless of a trauma history.

**Conclusion:**

Multipartite medial epicondyles occur in patients with current or previous elbow fractures only, whereas multipartite lateral epicondyles may be constitutional. Elbow fractures increase the prevalence of multipartite epicondyles on both sides, with significant lateral predominance.

**Key Points:**

• *Multipartite medial epicondyles should be considered of traumatic origin.*

*• Multipartite lateral epicondyles may be constitutional.*

*• Elbow fractures increase the prevalence of multipartite epicondyles on both sides with lateral predominance.*

## Introduction

**Fig. 1:**
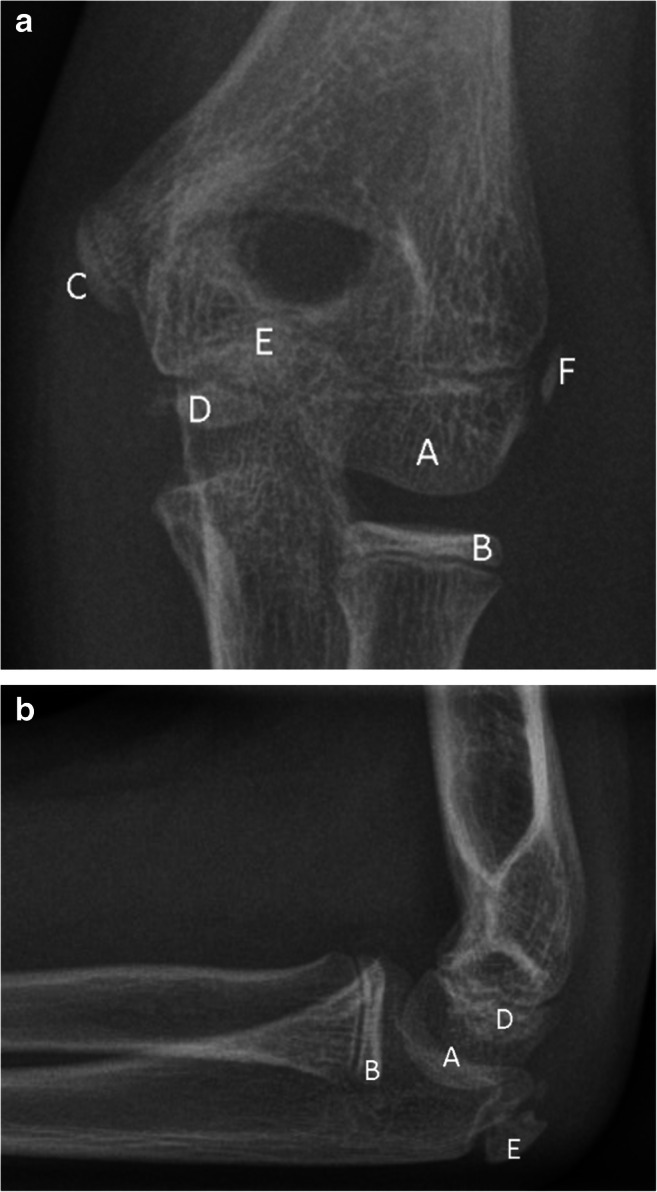
Normal radiograph of the left elbow of a 10-year-old girl in (**a**) anteroposterior projection and (**b**) lateral projection showing the six apophyses of the elbow joint: (A) capitellum, (B) radial head, (C) internal epicondyle, (D) trochlea, (E) olecranon, and (F) external epicondyle

Radiographs are the primary diagnostic imaging modality to rule out fractures in children after elbow trauma [1]. The evaluation of potential elbow fractures is complicated by the occurrence of several apophyses at specific times during skeletal development (Fig. 1) [2]. The timing of their occurrence varies individually, but follows a strict sequence that is described by the acronym CRITOE (capitellum, radial head, internal epicondyle, trochlea, olecranon, external epicondyle), although variances of this order in the minority of children has been described [3, 4]. Apophyseal fusion follows a slightly different order, indicated by the acronym CTE-R-O-I, which has been used in a recent publication by Kunc et al [5]. First, the capitellum, trochlea, and external epicondyle fuse together before fusing with the humerus. Later, the radius, olecranon, and internal epicondyle fuse [3]. Precise knowledge of the location and usual configuration of the six apophyses is necessary to identify potential fractures, avulsions, or apophyseal dislocations.

**Fig. 2:**
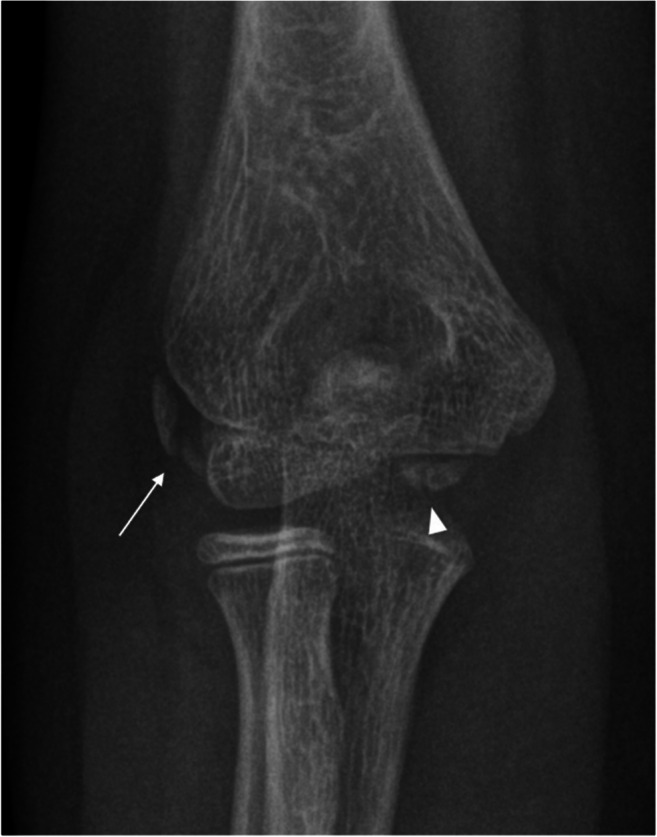
Radiograph of the right elbow of a 10-year-old boy in anteroposterior projection showing a multipartite lateral epicondyle consisting of two portions (arrow). The trochlear apophysis is typically multipartite and in this case consists of at least two parts (arrowhead)

Some apophyses may be multipartite, representing normal variants of skeletal development, as shown in Fig. 2. Those ossification centers may persist as it has been described for the olecranon and the epicondyles as physiological variants that should not be confused with avulsion fractures [4, 5]. Multipartition of the epicondyles can also be mimicked by linear or curved ossifications adjacent to the epicondyles as a result of posttraumatic soft tissue calcification, similar to the Stieda-Pellegrini lesion of the knee [6] or by apophyseal fragmentation in severe cases of apophysitis in children with repeated valgus stress (little leaguers’ elbow) [7].

Knowledge of normal variants of skeletal development is of utmost importance to detect or rule out fractures with a high degree of confidence, especially when the quality of radiographs is compromised by the limited compliance of children in a trauma situation. To date, no studies have specifically addressed multipartite medial and lateral epicondyles in the setting of pediatric elbow trauma. The purpose of this work was to investigate the prevalence of a multipartite appearance of the epicondyles and to assess its association with current or previous elbow fractures in pediatric patients undergoing diagnostic radiography.

## Materials and methods

### Study design

This retrospective single-center study examined the prevalence of multipartite epicondyles during skeletal development and their association with elbow fractures in a pediatric study population. The following null hypotheses were tested:

- H0_1_:There is no difference in the prevalence of multipartite medial and lateral epicondyles.
- H0_2_:There is no association between multipartite medial and lateral epicondyles and current or previous elbow fractures.
- H0_3:_ There is no difference in the prevalence of multipartite medial and lateral epicondyles between boys and girls regardless of current or past elbow fracture.

### Study population and data analyses

**Fig. 3:**
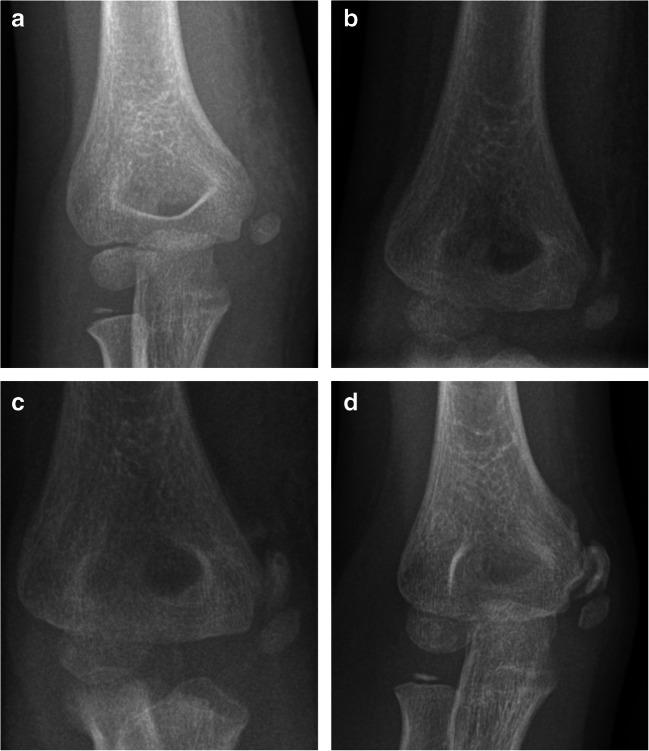
Radiographs of the right elbow of a 7-year-old girl in anteroposterior projection after elbow trauma. **a** Initial radiograph showing the ossification center of the medial epicondyle located somewhat distant from the humerus, consistent with an avulsion of the epicondyle. **b** The follow-up radiograph after 7 days shows a half-moon-shaped ossification zone between the humerus and the still distant medial epicondyle leading to a multipartite appearance of the medial epicondyle. **c** Increasing size of the now ovoid callus next to the ossification center of the medial epicondyle after 14 days. **d** After 4 months, the posttraumatic medial epicondyle remains a multipartite appearance with further progression of the callus

Ethical approval for this retrospective study was waived by the institutional ethics committee. In this study, 4282 radiographs of 1265 elbows of 1210 patients aged 0 to 17 years, acquired between 2017 and 2019, were reviewed. In 36 boys and 19 girls, radiographs of both sides were included. The difference between the numbers of radiographs and the number of patients included results from follow-up radiographs. Only radiographs obtained in a trauma context were considered. In addition to dedicated elbow projections, also radiographs of the humerus and the forearm were included into the analyses if they covered the entire elbow joint. The elbow radiographs were analyzed in consensus reading by two radiologists, one with 9 years of musculoskeletal imaging experience and special certification as a pediatric radiologist and one with 2 years of musculoskeletal imaging experience, for presence of multipartite epicondyles. If an epicondyle was found to be multipartite, a review of previous radiographs (if present) and/or follow-up examinations (to exclude later fracture demarcation or development of indirect fracture signs) was performed to determine whether the multipartite appearance was associated with a previous or present elbow fracture (Fig. 3). If present, fractures of any type and location were noted and grouped as follows: Fractures of the proximal and middle third of the humerus as well as fractures of the middle and distal third of the forearm were classified as fractures not involving the elbow joint (thus not supposed to have any impact on epicondyle ossification). Fractures of the distal humerus and proximal forearm were classified as fractures involving the elbow joint. Direct fractures or avulsions of the epicondyles were grouped separately.

**Fig. 4:**
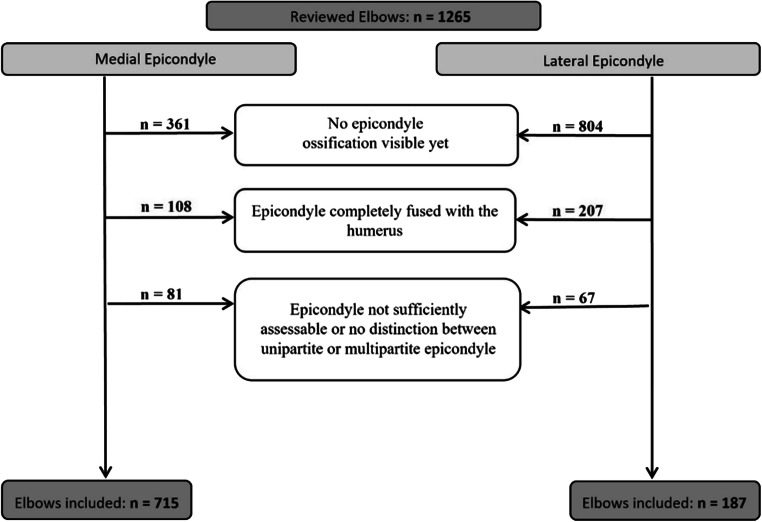
The flowchart shows the number of patients included, the exclusion criteria, and number of participants excluded, respectively. A separate study group was built for the analyses of each epicondyle

Separate study populations were built for the analyses of the medial and the lateral epicondyles. Radiographs that did not allow for the epicondyle to be examined with sufficient diagnostic quality to assess multipartition, e.g., due to a misprojection or osteosynthesis materials, were excluded from the analyses. If the examined epicondyle was not yet visible or already fused to the distal humerus, the corresponding radiograph was also excluded. Reviewed cases and excluded elbows are summarized in Fig. 4. The resulting study populations for analyses of the medial and lateral epicondyles are characterized in Table 1.

**Table 1:** Study population

	Medial epicondyle	Lateral epicondyle
Patients (*n*)		
Total	689	183
Female	329 (47.8%)	94 (51.4%)
Male	360 (52.2%)	89 (48.6%)
Elbows (*n*)^1^		
Total	715	187
Female	339 (47.4%)	96 (51.3%)
Male	376 (52.6%)	91 (48.7%)
Age (years)		
Total	8.9 ± 3.0 (1–17)	10.7 ± 1.8 (1–17)
Female	7.8 ± 2.8 (1–14)	9.7 ± 1.3 (6–14)
Male	9.9 ± 2.9 (1–17)	11.8 ± 1.7 (7–17)

^1^The difference between the number of patients and the number of elbows included results from patients in whom radiographs of both elbows were obtained during the observation period

A total of 715 elbows was included for analyses of the medial epicondyle. Of these, 225 extremities had no fracture, 211 had a humerus or forearm fracture distant from the elbow joint, 255 had a fracture involving the elbow joint but without involvement of the medial epicondyle, and 24 had a direct fracture or avulsion of the medial epicondyle. A total 187 elbows was included for lateral epicondyle analyses. Of these, 79 extremities had no fracture, 38 had a humeral or forearm fracture distant from the elbow joint, 64 had a fracture including the elbow joint but without involvement of the lateral epicondyle, and 6 had a fracture or avulsion of the lateral epicondyle. The significantly larger number of elbows included for medial epicondyle analysis results from the fact that the ossification center of the medial epicondyle appears earlier in skeletal development and fuses later with the humerus when compared to the lateral epicondyle [2, 4].

### Statistical analyses

Statistical tests were performed by using dedicated software (SPSS version 25; IBM and GraphPad Prism version 9.2.0 for Windows; GraphPad Software, Inc.). Descriptive statistics were used to assess the frequencies of multipartite epicondyles throughout the study groups. The prevalence of multipartite lateral and medial epicondyles and their prevalence in patients with and without elbow fractures were compared using the chi-square test. Odds ratios (OR) were computed to compare the relative odds of the occurrence of multipartite medial and lateral epicondyles, given exposure to the variables of interest. The Haldane-Anscombe correction was used to calculate the OR when the observed frequency of multipartition in a given study group was zero. Examined variables were “elbow fracture” and “gender.” Ninety-five percent confidence intervals are given in square brackets. *p* values < 0.05 were considered statistically significant.

## Results

### Prevalence of multipartite epicondyles

Multipartite medial epicondyles occurred exclusively in patients with current or previous elbow fractures. None of the patients without any upper extremity fracture (*n* = 225) or an upper extremity fracture distant to the elbow joint (*n* = 211) had a multipartite medial epicondyle. In contrast, multipartite lateral epicondyles did occur in patients with and without current or previous elbow fractures. Patients without any history of an upper extremity fracture had a multipartite lateral epicondyle in 8% (*n* = 6 of 79). The difference in prevalence of multipartite medial and lateral epicondyles was highly significant in patients without any history of an elbow fracture (*p* < 0.001). Including all patients regardless of elbow fracture history, multipartite medial epicondyles were observed in 3% (*n* = 24 of 715) and multipartite lateral epicondyles in 18% (*n* = 33 of 187). This difference in prevalence was highly significant as well (*p* < 0.001).

### Association of multipartite epicondyles and upper extremity fractures

Multipartite medial and lateral epicondyles occurred significantly more often in patients with elbow fractures of any kind than without elbow fractures, with an exception in patients with lateral epicondyle fractures, in whom the difference was no longer significant. The absolute numbers and frequencies of multipartite epicondyles in pediatric patients with and without upper extremity fractures of different types are summarized in Table 2.

**Table 2:** Absolute numbers and frequencies of multipartite medial and lateral epicondyles in patients with and without current or previous upper extremity fractures

		Lateral epicondyle					Medial epicondyle				
		All (*n*)	Multipartite (*n*)	%	*p*	OR	All (*n*)	Multipartite (*n*)	%	*p*	OR
1	No fracture	79	6	8			225	0	0		
2	Fracture distant from the elbow joint	38	4	11			211	0	0		
1 + 2	No fracture and fracture distant from elbow joint	117	10	9			436	0	0		
3	Elbow fracture without epicondyle involvement	64	21	33	< 0.001	5 [2;12]	255	15	6	< 0.001	56 [3;945]
4	Elbow fracture with epicondyle involvement	6	2	33	> 0.05	5 [1;33]	24	9	38	< 0.001	535 [30;9615]
3 + 4	Elbow fracture with and without epicondyle involvement	70	23	33	< 0.001	5 [2;12]	279	24	9	< 0.001	84 [5;1383]

*p* values (*p*) and odds ratios (OR) result from the chi-square test. Comparisons were made between the groups 3, 4, 3 + 4, and the group 1 + 2

### Comparison of the medial and lateral epicondyles

In patients with elbow fractures excluding epicondyle involvement, multipartite epicondyles were observed medially in 6% (*n* = 15 of 255) and laterally in 33% (*n* = 21 of 64), being a highly significant difference (*p* < 0.001). Focusing on elbow fractures with epicondyle involvement only, this difference was 38% medially (*n* = 9 of 24) vs. 33% laterally (*n* = 2 of 6) and no longer significant (*p* > 0.05). Summarizing all elbow fractures with and without epicondyle involvement, multipartite epicondyles were observed in 9% medially (*n* = 24 of 279) vs. 33% laterally (*n* = 23 of 70) (*p* < 0.001).

### Gender differences in the prevalence and fracture association of multipartite epicondyles

No significant differences between girls and boys were observed regarding prevalence and fracture association of multipartite epicondyles. Table 3 summarizes the distribution of multipartite medial and lateral epicondyles by age and gender. The majority of multipartite medial epicondyles occur between 6 and 9 years and the majority of lateral epicondyles between 9 and 11 years, matching the delayed onset of lateral epicondyle ossification according to the aforementioned CRITOE acronym. Table 4 shows the absolute numbers and percentages of multipartite medial and lateral epicondyles in boys and girls with and without elbow fractures.

**Table 3:** Absolute numbers of multipartite medial and lateral epicondyles sorted by age and gender

	Multipartite medial epicondyle (*n* = 24)				Multipartite lateral epicondyle (*n* = 33)			
Age (years)	No fracture	Elbow distant fracture	Elbow fracture excluding medial epicondyle	Medial epicondyle fracture	No fracture	Elbow distant fracture	Elbow fracture excluding lateral epicondyle	Lateral epicondyle fracture
4	–	–	2 (1 f,1 m)	–	–	–	–	–
5	–	–	–	–	–	–	–	–
6	–	–	4 (1 f, 3 m)	1 (1 f)	–	–	1 (1 f)	–
7	–	–	1 (1 m)	2 (1 f, 1 m)	–	–	1 (1 m)	–
8	–	–	3 (1 f, 2 m)	–	1 (1 f)	–	1 (1 f)	–
9	–	–	4 (1 f, 3 m)	2 (2 f)	1 (1 f)	–	7 (2 f, 5 m)	–
10	–	–	–	–	2 (1 f, 1 m)	2f	5 (4 f, 1 m)	1 (1 m)
11	–	–	–	1 (1 f)	2 (2 m)	–	3 (2 f, 1 m)	1 (1 f)
12	–	–	–	2 (1 f, 1 m)	–	1 (1 m)	1 (1 m)	–
13	–	–	1 (1 m)	1 (1 m)	–	1 (1 m)	–	–
14	–	–	–	–	–	–	2 (1 f, 1 m)	–

*f* female, *m* male

**Table 4:** Absolute numbers and percentages of multipartite medial and lateral epicondyles with and without elbow fractures, divided into boys and girls

	Medial epicondyle								Lateral epicondyle							
	Girls			Boys			*p*	OR	Girls			Boys			*p*	OR
	All (*n*)	Multipartite (*n*)	%	All (*n*)	Multipartite (*n*)	%			All (*n*)	Multipartite (*n*)	%	All (*n*)	Multipartite (*n*)	%		
No elbow fracture	0	0	–	0	0	–	–	–	59	5	8	58	5	9	> 0.05	1
Elbow fracture	138	10	7	141	14	10	> 0.05	1	37	12	32	33	11	33	> 0.05	1
All patients	339	10	3	376	14	4	> 0.05	1	96	17	18	91	16	18	> 0.05	1

*p* values (*p*) and odds ratios (OR) result from the chi-square test

## Discussion

The purpose of this study was to investigate the prevalence of multipartite epicondyles during skeletal development and their association with pediatric elbow fractures. The prevalence of multipartition was significantly higher for the lateral epicondyle in both patients with and without current or previous upper extremity fracture. Multipartite medial epicondyles were observed in association with elbow fractures only, whereas multipartite lateral epicondyles occurred in 9% of cases without any evidence of an elbow fracture. Consequently, the null hypothesis H0_1_ that there is no difference in the prevalence of multipartite medial and lateral epicondyles could be rejected. A history of elbow fracture significantly increased the prevalence of multipartite epicondyles on both sides with lateral predominance. In fractures directly involving the epicondyles, the prevalence of multipartition was significantly increased on both sides, but without significant predominance of either side. Thus, the null hypothesis H0_2_ that there is no association between multipartite medial and lateral epicondyles and current or past elbow fractures could also be rejected. In our study population, there was no significant difference in the prevalence of multipartite epicondyles between boys and girls, regardless of upper extremity fractures. Consequently, the null hypothesis H0_3_ that there is no association between the multipartition of the medial and lateral epicondyles and gender was confirmed.

Although it is assumed that epicondylar ossification usually originates from a single ossification center [8], both multipartite medial and lateral epicondyles have been described before [9], as well as the occurrence of accessory ossicles of the medial and lateral epicondyles in adults [10]. A recently published study assessing the prevalence of accessory bones of the adult elbow mentions the rare occurrence of accessory ossicles or sesamoid bones next to the medial epicondyle in 0.46% and lateral epicondyle in 0.21%, which is significantly lower than the prevalence of multipartite epicondyle ossification in this study [5]. Consequently, it can be assumed that multipartite epicondyles fuse into one epicondyle in the majority of cases and that remaining accessory ossicles in adulthood are an exception. To date, there have been no dedicated analyses of the prevalence of multipartite epicondyles in children with and without a history of elbow or epicondyle fracture. Isolated fractures of the lateral epicondyle appear to be rare, but lateral condylar fractures are the second most common elbow fracture after supracondylar fractures and may affect epicondylar ossification [11]. Fractures of the medial epicondyle represent the third most common type of elbow fracture in children and may cause multipartition of the medial epicondyle [11]. Thus, the results of the present analysis expand the available evidence on the prevalence of multipartite epicondyles and may help to distinguish traumatic epicondyle injuries from atraumatic developmental variants of epicondyle ossification in pediatric radiographs.

Based on the findings of this study, a multipartite medial epicondyle must be considered traumatic in origin, whereas a multipartite lateral epicondyle can be both traumatic and an atraumatic developmental variant. The detection and appropriate treatment of medial epicondylar fractures, mostly caused by a posterior elbow luxation or valgus trauma [12], and the distinction between exclusively epicondylar fractures and unstable fractures involving the medial condyle, are important for a good functional outcome and reduction of posttraumatic complications, such as stiffness, instability, deformity, symptomatic nonunion, articular incarceration of the fracture fragment, or ulnar nerve injury [15, 16]. The detection of medial epicondylar fractures in radiographs may be difficult due to anatomic reasons, as the medial epicondyle is located outside the joint capsule and a fracture limited to this epicondyle will usually not result in a positive fat pad sign [13]. In a recent study, Cao et al suggested to additionally perform an axial radiograph of the elbow for a more accurate determination of medial epicondyle displacement in patients with medial epicondyle fractures [14]. Accordingly, failure to recognize and appropriately treat unstable lateral condylar fractures can lead to posttraumatic complications, e.g., nonunion, cubitus valgus, ulnar nerve injury, or stiffness [15]. The increased prevalence of a multipartite lateral epicondyle in patients with an elbow fracture may give rise to fracture suspicion and warrant radiographic follow-up to rule out the development of fracture-specific alterations at a later time. Of course, clinical symptoms should be included in this decision-making process.

### Study limitations

The analyzed radiographs were taken in the context of upper extremity trauma to rule out fracture. Therefore, in individual cases, an atraumatic cause for a multipartite epicondyle cannot be excluded with absolute certainty. Also, it cannot be completely ruled out that a particular multipartite epicondyle may have resulted from a previous fracture. To minimize these false-negative findings related to fracture association of a multipartite epicondyle, previous radiographs (if available) and/or follow-up examinations were reviewed to determine with best possible certainty whether a specific multipartite epicondyle was associated with an elbow fracture. Because multipartite medial epicondyles were not observed in patients without a history of elbow fracture, the Haldane-Anscombe correction was required to calculate the OR, resulting in typically wide confidence intervals.

## Conclusion

A multipartite medial epicondyle must be considered traumatic in origin, whereas a multipartite lateral epicondyle can be both traumatic and an atraumatic developmental variant. The strong increase in prevalence of multipartite lateral epicondyles in patients with elbow fracture should raise suspicion for fracture when a multipartite lateral epicondyle is detected and may warrant radiographic follow-up.

## Abbreviations

CRITOE: Capitellum, radial head, internal epicondyle, trochlea, olecranon, external epicondyle
CTE-R-O-I: Capitellum, trochlea, external epicondyle, radial head, olecranon, internal epicondyle

## Declarations

### Guarantor

The scientific guarantor of this publication is Andrea Schmid.

### Statistics and Biometry

No complex statistical operations were used in this manuscript.

### Methodology

• Retrospective

• Observational

• Performed at one institution
